# Mechanism for Higher Tolerance to and Lower Accumulation of Arsenite in *NtCyc07*-Overexpressing Tobacco

**DOI:** 10.3390/plants9111480

**Published:** 2020-11-03

**Authors:** DongGwan Kim, Ramin Bahmani, Mahsa Modareszadeh, Seongbin Hwang

**Affiliations:** Department of Bioindustry and Bioresource Engineering, Department of Molecular Biology and Plant Engineering Research Institute, Sejong University, Seoul 05006, Korea; kimdg@sejong.ac.kr (D.K.); rbahmani@snu.ac.kr (R.B.); mmodareszadeh@sju.ac.kr (M.M.)

**Keywords:** aquaporin, arsenic, Cyc07, NIP, PIP, ROS, tobacco

## Abstract

Arsenite [As(III)] is a highly toxic chemical to all organisms. Previously, we reported that the overexpression of *NtCyc07* enhanced As(III) tolerance and reduced As(III) accumulation in yeast (*Saccharomyces cerevisiae*) and tobacco (*Nicotiana tabacum*). To understand a mechanism for higher As(III) tolerance and lower As(III) accumulation in *NtCyc07*-overexpressing tobacco, we examined the expression levels of various putative As(III) transporters (aquaporin). The expressions of putative As(III) exporter *NIP1;1*, *PIP1;1*, *1;5*, *2;1*, *2;2*, and *2;7* were enhanced, while the expressions of putative As(III) importer *NIP3;1*, *4;1*, and *XIP2;1* were decreased, contributing to the reduced accumulation of As(III) in *NtCyc07*-overexpressing tobacco. In addition, the levels of oxidative stress indicators (H_2_O_2_, superoxide and malondialdehyde) were lower, and the activities of antioxidant enzymes (catalase, superoxide dismutase and glutathione reductase) were higher in *NtCyc07*-tobacco than in the control tobacco. This suggests that the lower oxidative stress in transgenic tobacco may be attributed to the higher activities of antioxidant enzymes and lower As(III) levels. Taken together, the overexpression of *NtCyc07* enhances As(III) tolerance by reducing As(III) accumulation through modulation of expressions of putative As(III) transporters in tobacco.

## 1. Introduction

Arsenic is present in the land, air and ground water, and acts as a toxic substance to humans, animals and plants [1]. In most cases, arsenic enters humans through drinking water or crops which absorb arsenic that dissolves in rainwater and flows into the environment [2]. The concentration that does not harm humans is less than 10~50 ppb [3], and over than this concentration, the acute and chronic arsenic toxicity will appear [4]. The main toxic effects to humans are cancer [5,6], heart disease (hypertension-related cardiovascular disease) [7], stroke (cerebrovascular diseases) [8], chronic respiratory disease [9] and diabetes [10].

Major forms of inorganic arsenic in the environment, As(III) (arsenite) and As(V) (arsenate), can enter plants through high-affinity Pi transporters and the Nod26-like Intrinsic Protein (NIP) subfamily of aquaporins, respectively [11,12,13]. Plant aquaporins can be categorized into four subgroups: Plasma membrane Intrinsic Proteins (PIPs), Nod26-like Intrinsic Proteins (NIPs), Tonoplast Intrinsic Proteins (TIPs) and Small Basic Intrinsic Proteins (SIPs). The NIPs are localized at plasma/intracellular membranes, while SIPs are at the endoplasmic reticulum [14]. Aquaporins participate in transportation of water, ammonia, arsenic, boric acid, glycerol, metalloids, nitric oxide, and reactive oxygen species [15,16,17,18,19,20]. All reported NIPs have As(III) influx activities [11,13,21,22,23,24,25,26]; the knockout *Arabidopsis* of *AtNIP1;1* and *AtNIP7;1* displayed higher tolerance to and lower accumulation of As(III) [13,27]. The *nip1;2* and *nip5;1* mutants of *Arabidopsis* accumulated less As(III), while they did not show a higher tolerance to As(III) [27]. In addition, *OsNIP3;2* is involved in As(III) uptake in rice since a knockout mutant showed lower accumulations of As(III) in its roots, compared to WT plants [28]. It has been reported that, among *NIPs*, *AtNIP5;1* and *AtNIP6;1* of *Arabidopsis*, *OsNIP2;1* (*OsLsi1*) and *OsNIP3;2* of rice, and *LjNIP5;1* and *LjNIP6;1* of *Lotus japonicus* are involved in the bi-directional transport (both uptake and export) of As(III) in yeast or plants [11]. Among PIPs, the overexpression of *OsPIP2;4, OsPIP2;6* and *OsPIP2;7* enhanced As(III) tolerances in Arabidopsis. Interestingly, *OsPIP2;6*-expressing *Arabidopsis* exhibited higher activities in As(III) uptake and export in roots in response to short-term (4 h) exposure to As(III) [29]. Recently, 88 aquaporin genes were identified in *Nicotiana tabacum* genome and assigned into five subfamilies: 34 plasma membrane intrinsic proteins (PIPs); 27 tonoplast intrinsic proteins (TIPs); 20 nodulin26-like intrinsic proteins (NIPs); three small basic intrinsic proteins (SIPs); four uncharacterized X intrinsic proteins (XIPs) [30]. 

The *Cyc07* gene was first reported in *Catharanthus roseus* [31]. *Cyc07* mRNA was detected specifically in the S phase of synchronous cultures as well as in intact plants, and was found mainly in the meristem zone of root tips [32]. Cyc07 has a domain of ribosomal S3Ae [32]. Tobacco Cyc07 (NtCyc07) was cloned and identified to be involved in arsenite tolerance by reducing As accumulation through interacting with ACR1 which is a transcription factor of the arsenite exporter ACR3 in yeast [33]. In addition, the overexpression of *NtCyc07* in tobacco enhanced arsenite tolerances by decreasing As accumulation [34]. Tobacco has a single gene of NtCyc07. Regarding genes involved in arsenic tolerance in tobacco, it was only reported that the overexpression of tobacco phytochelatin synthase (NtPCS1) enhances arsenite tolerances in yeast [35] and tobacco [36].

In this report, to understand a mechanism for higher tolerance to and lower accumulation of As(III) in *NtCyc07*-expressing tobacco, we have examined expressions of putative As(III) transporters *NIPs* and *PIPs*, ROS (reactive oxygen species) levels and antioxidant enzyme activities. We found that NtCyc07 enhanced As(III) tolerances by decreasing As(III) accumulations through regulation of expressions of putative As(III) transporters. In addition, *NtCyc07*-tobacco displayed a lower oxidative stress and higher activities of antioxidant enzymes, which may be ascribed to the lower accumulation of As(III).

## 2. Results and Discussion

### 2.1. NtCyc07-Overexpressing Tobacco Displayed Higher Tolerance to and Lower Accumulation of As(III)

**Figure 1:**
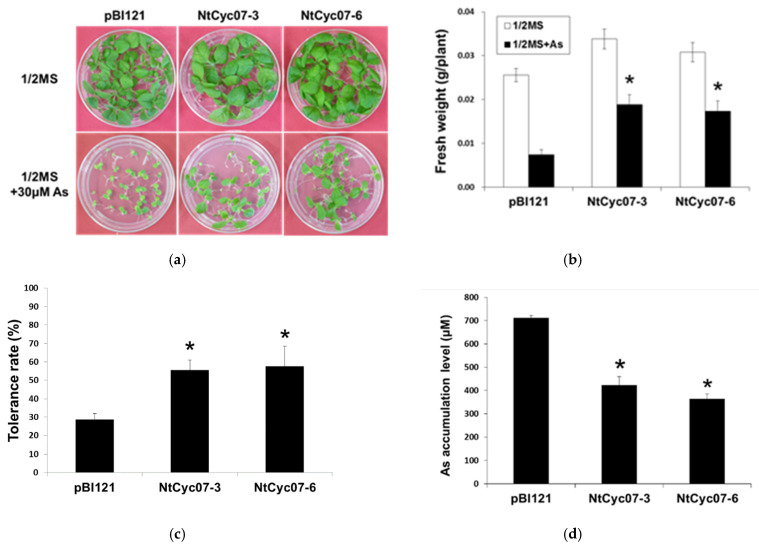
As(III) tolerance and level in *NtCyc07*-expressing tobacco. (**a**) Comparison of morphology and As(III) tolerance in transgenic tobacco expressing *pBI121* only (control) and *NtCyc07* (NtCyc07-3 and NtCyc07-6) in response to As(III) stress. All plants were germinated and grown for 3 weeks on 1/2 Murashige and Skoog medium (MS) agar plates without (upper) and with (lower) 30 µM As(III). (**b**) Fresh weights of tobacco shown in (**a**). (**c**) As(III) tolerance rates of tobacco shown in (**a**,**b**). (**d**) As(III) accumulations in whole seedlings of control and *NtCyc07* tobacco shown in (**a**). 1/2 MS indicates a control (no As(III) treatment). Each value corresponds to the means of three independent experiments, and error bars indicate standard errors. Asterisks indicate significant differences between control and transgenic tobacco (*p* ≤ 0.05).

We have examined again As(III) tolerance and accumulation in a vector only expressing control (pBI121) and *NtCyc07*-overexpressing (*NtCyc07-3* and *NtCyc07-6*) tobacco since we challenged plants with 30 µM As(III) which is higher than previously used 15 µM [34] to induce a remarkable difference in As(III) tolerance between control and transgenic plants. As shown in Figure 1a,b, *NtCyc7*-tobacco exhibited a higher tolerance to As(III) than control plants, and control and transgenic plants were healthy based on green colors/chlorophylls even with enhanced As(III) concentrations. In addition, As(III) accumulation was lower in *NtCyc07*-tobacco than in control plants (Figure 1c), suggesting that the higher As(III) tolerance in transgenic tobacco is ascribed to the reduced level of As(III). 

Supporting our results, many studies have reported that the higher metal tolerance is ascribed to the lower accumulation of metal in plants. Indian mustard (*Brassica juncea*) with moderate expression of *AtPCS1* (Phytochelatin Synthase 1 of *Arabidopsis*) exhibited increased tolerance to and reduced accumulation of Cd and Zn [37]. Overexpression of tobacco *Cyc07* (cell cycle-related protein 7) enhanced As(III) tolerance and reduced As(III) accumulation in tobacco [34]. The overexpression of tobacco *PIC1* (Permease In Chloroplast 1) in tobacco enhanced Cd tolerance and reduced Cd content [38]. Overexpression of tobacco *Hb1* (non-symbiotic class 1 hemoglobin 1) increased Cd tolerance and decreased Cd level in tobacco [39] and Arabidopsis [40]. The overexpression of *CsMTP8* (Metal Transport Protein 8 of tea plant *Camellia sinensis)* enhanced Mn tolerance and reduced Mn accumulation in Arabidopsis [41]. Transgenic tobacco overexpressing tobacco *UBC1* (ubiquitin-conjugating protein 1) displayed higher tolerance to and lower level of Cd [42]. Overexpression of *WRKY13* enhanced a Cd tolerance and reduced Cd accumulation by increasing the expression of Cd exporter PDR8 [43]. Transgenic rice which expresses *TaCNR2* (Cell Number Regulator 2 of wheat *Triticum aestivum*) showed an enhanced Cd tolerance and a reduced Cd level [44]. Overexpression of tobacco *UBQ2* (ubquitin-extension protein 2) in tobacco and *Arabidopsis* increased a Cd tolerance and reduced a Cd level [45]. Transgenic *Arabidopsis* overexpressing miRNA156 displayed higher Cd tolerance and lower Cd level [46].

### 2.2. Expressions of Putative As(III) Transporters Were Modulated in NtCyc07-Tobacco

Previously, we have reported that *NtCyc07*-overxpressing tobacco displayed a higher As(III) tolerance and the lower accumulation of As(III), compared with control plants [34]. To understand a molecular mechanism for the higher As(III) tolerance and lower accumulation of As(III) in *NtCyc07*-tobacco, the expression level of putative As(III) transporter aquaporin including *NtNIP1;1* (XP_016487110.1), *NtNIP2;1* (XP_016451246.1), *NtNIP3;1* (XP_016460638.1), *NtNIP4;1* (XP_016486634.1), *NtPIP1;1* (NP_001313131.1), *NtPIP1;5* (AAB81601.1), *NtPIP2;1* (AF440272.1), *NtPIP2;2* (NM_001325404.1), *NtPIP2;7* (NP_001313061.1), *NtPIP2;17* (NP_001312464.1), *NtXIP1;1α* (NP_001312796) and *NtXIP2;1* (XP_016489264.1), were examined. To date, the involvement of aquaporin in the As(III) transport has never been reported in tobacco. Therefore, the putative As(III) transporters were selected among NIPs and PIPs based on protein sequences showing differences in homology, substrate specificity determining positions (SDPs), and plasma membrane localization [30]. 

**Figure 2:**
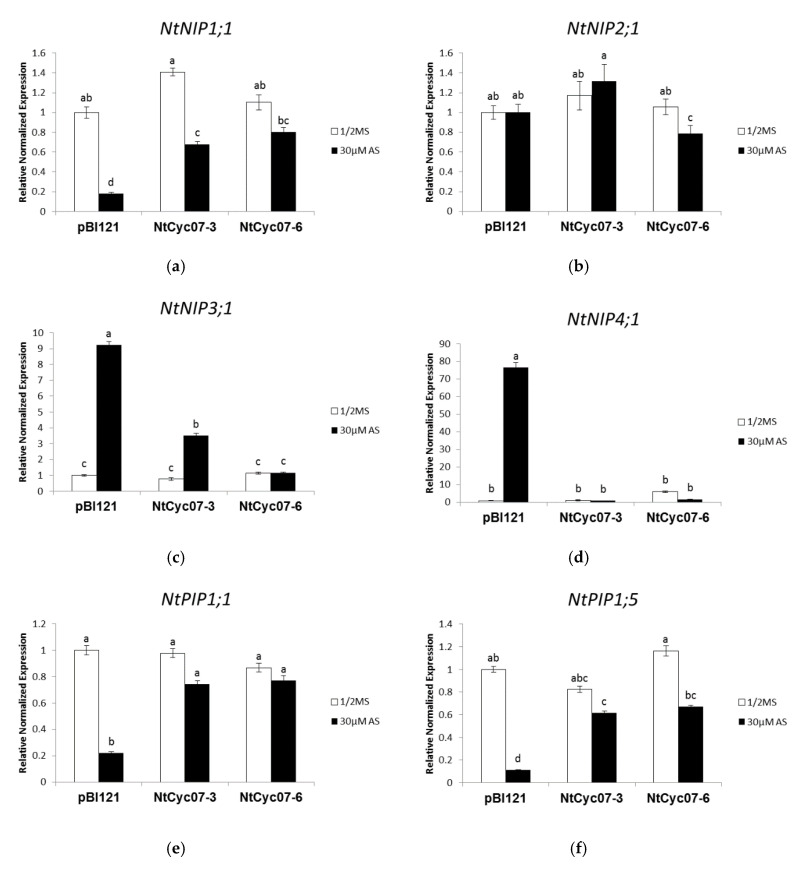

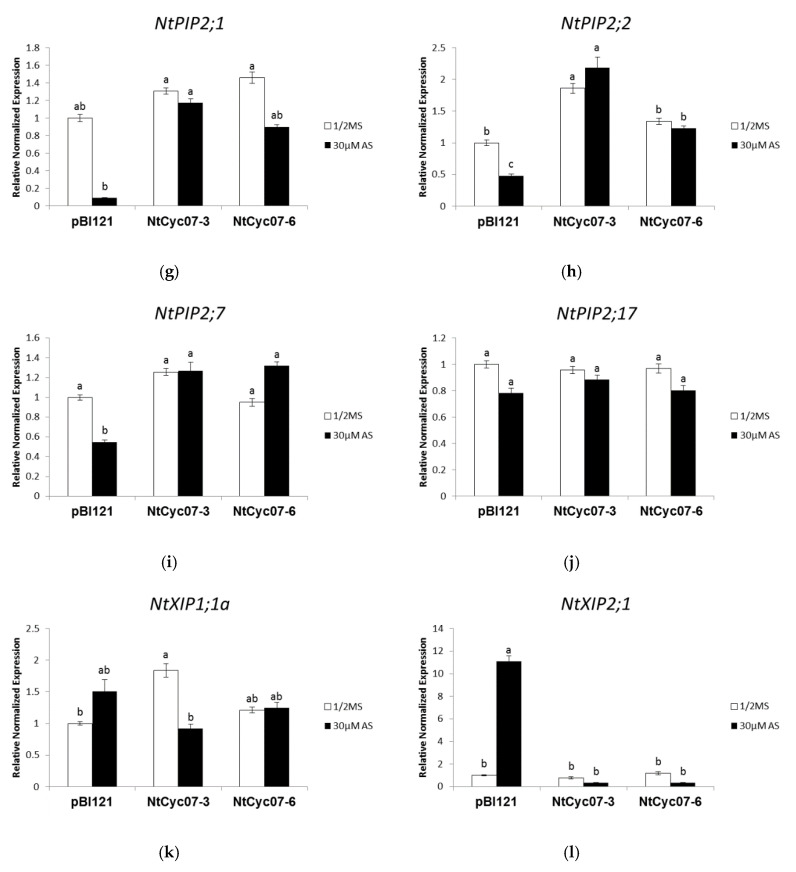
Relative expression levels (in quantitative RT-PCR—qRT-PCR) of As(III) transporters in *NtCyc07*-tobacco. qRT-PCR analysis showing the expression levels of (**a**) *NtNIP1;1* (XP_016487110.1 ), (**b**) *NtNIP2;1* (XP_016451246.1), (**c**) *NtNIP3;1* (XP_016460638.1), (**d**) *NtNIP4;1* (XP_016486634.1), (**e**) *NtPIP1;1* (NP_001313131.1), (**f**) *NtPIP1;5* (AAB81601.1), (**g**) *NtPIP2;1* (AF440272.1), (**h**) *NtPIP2;2* (NM_001325404.1), (**i**) *NtPIP2;7* (NP_001313061.1), (**j**) *NtPIP2;17* (NP_001312464.1), (**k**) *NtXIP1;1α* (NP_001312796) and (**l**) *NtXIP2;1* (XP_016489264.1) in control (*pBI121*) and transgenic (*NtCyc07*) tobacco. Total RNA was isolated from tobacco seedlings grown for 3 weeks on 1/2 MS agar media supplemented without or with 30 µM sodium arsenite. 1/2 MS indicates a control (no As(III) treatment). The data are averages of three independent experiments per each treatment, and error bars indicate standard errors (S.E.). Different letters over columns indicate significant differences (*p* ≤ 0.05) between treatments.

As shown in Figure 2a–d, among NIPs, *NIP1;1* expression was higher and expressions of *NIP3;1* and *NIP4;1* were lower in *NtCyc07*-tobacco than in control plants, while the expression level of *NIP2;1* was not different from that of control. This suggests that NIP1;1, NIP3;1 and NIP4;1 may be involved in the lower accumulation of As(III) in *NtCyc07*-expressing tobacco. In support of this, SDP data show that NIP1;1, NIP3;1 and NIP4;1 had a As specificity but NIP2;1 did not [30]. Based on these data, it is assumed that NIP1;1 has a higher activity of As(III) exporter than of As(III) importer, while NIP3;1 and NIP4;1 has a higher activity of As(III) importer. Therefore, the modulated expressions of these aquaporins may contribute to the reduced accumulation of As(III) in transgenic tobacco.

As shown in Figure 2e–j, among PIPs which do not have As specificity based on SDP [30], expressions of *PIP1;1*, *PIP1;5*, *PIP2;1*, *PIP2;2*, and *PIP2;7* were higher in *NtCyc07*-tobacco than in control plants, while *PIP2;17* expression did not show a difference from that of control tobacco. Therefore, it is assumed that PIP1;1, PIP1;5, PIP2;1, PIP2;2, and PIP2;7 are involved in decreasing As(III) accumulation by higher activity of As(III) efflux in transgenic tobacco.

In addition, we examined expression levels of two XIP in response to As(III) since XIP was not characterized yet [30]. As shown in Figure 2k,l, *XIP1;1α* expression level in *NtCyc07*-tobacco was not different from that of control plants, while the expression level of *XIP2;1* was lower in transgenic tobacco than in control plants. This suggests that XIP2;1 may have a higher activity of As(III) influx, therefore its reduced expression contributes to the lower level of As(III) in *NtCyc07*-expressing tobacco. Taken together, although all aquaporins were not examined, the overexpression of *NtCyc07* enhances the expressions of putative As(III) exporter *NIP1;1, PIP1;1, PIP1;5, PIP2;1, PIP2;2, and PIP2;7,* while it reduces the expression of putative As(III) importer *NIP3;1, NIP4;1,* and *XIP2;1,* contributing to the reduced accumulation of As(III) in *NtCyc07*-overexpressing tobacco. However, gene expressions do not reflect exactly protein amounts and activities, thus it is possible that the involvement and role of presented transporters in As(III) transport may be different from our assumption. 

Regarding the involvement of aquaporin in As(III) transport, many NIPs and PIPs have been reported: all these have As(III) import activity, and some (AtNIP5;1, AtNIP6;1, OsNIP2;1, OsNIP3;2, LjNIP5;1, LiNIP6;1, OsPIP2;4, OsPIP2;6, and OsPIP2;7) have both import and export activities. In contrast, the participation of aquaporin in As(III) transport has never been reported in tobacco. Therefore, our study is the first report showing the role of aquaporin in As(III) transport in tobacco. However, until the As(III) import/export activity of tobacco NIPs and PIPs are experimentally proved using oocyte injection or thermodynamic kinetics of As uptake/efflux in plants, our data about roles of NIPs and PIPs in As(III) transport are indirect or assumptive.

### 2.3. NtCyc07-Expressing Tobacco Displayed a Lower Oxidative Stress and Higher Activities of Antioxidant Enzymes in Response to As(III)

**Figure 3:**
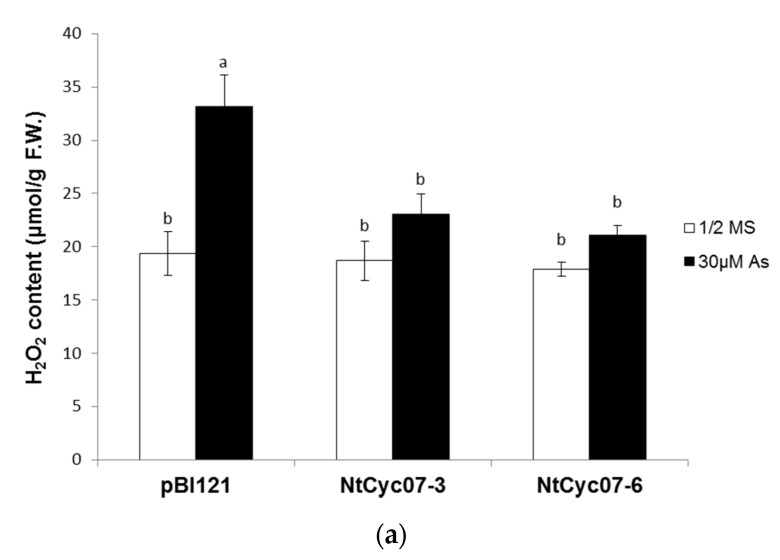

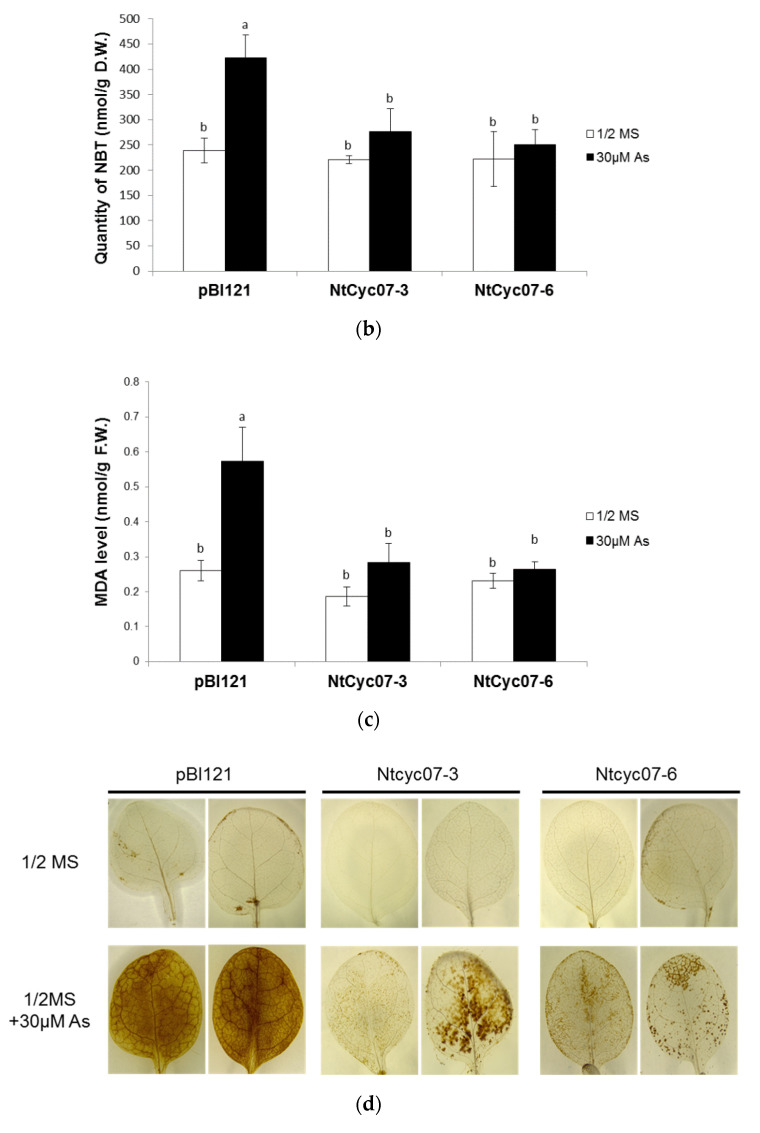

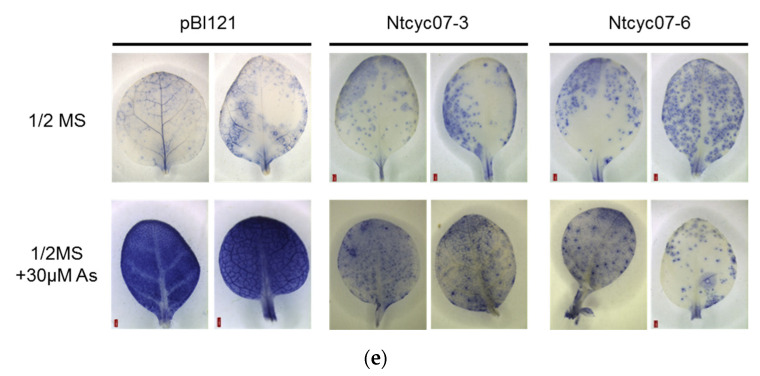
Oxidative stress levels of *NtCyc07*-overexpressing tobacco in response to As(III). Levels of (**a**) hydrogen peroxide, (**b**) superoxide, (**c**) malondialdehyde (MDA), (**d**) DAB (3,3-diaminobenzidine) staining for H_2_O_2_ and (**e**) nitro blue tetrazolium (NBT) staining for superoxide in control (pBI121) and *NtCyc07*-tobacco. Plants were germinated and grown for 3 weeks on 1/2 MS agar media supplemented without or with 30 µM As(III). 1/2 MS indicates a control (no As(III) treatment). The data are averages of three independent experiments per each treatment, and error bars indicate standard errors (S.E.). Different letters over columns indicate significant differences (*p* ≤ 0.05) between treatments.

Since As(III) treatments generally induce oxidative stress [47], we compared oxidative stress levels including hydrogen peroxide (H_2_O_2_), superoxide (O_2_^−^) and malondialdehyde (MDA, indicator of membrane oxidation) between control and *NtCyc07*-tobacco (Figure 3a–c). While levels of H_2_O_2_, superoxide and MDA were enhanced by As(III) in control tobacco, those were not altered in *NtCyc07*-tobacco, resulting in lower oxidative stress levels in transgenic tobacco than in control plants. In addition, levels of H_2_O_2_ and superoxide were visualized by DAB and NBT staining, respectively (Figure 3d,e). It also confirmed lower contents of H_2_O_2_ and superoxide in transgenic tobacco, compared with that of control plants. Considering the lower levels of As(III) and oxidative stress in *NtCyc07*-tobacco, the lower oxidative stress may be attributed to the lower As(III) level. 

**Figure 4:**
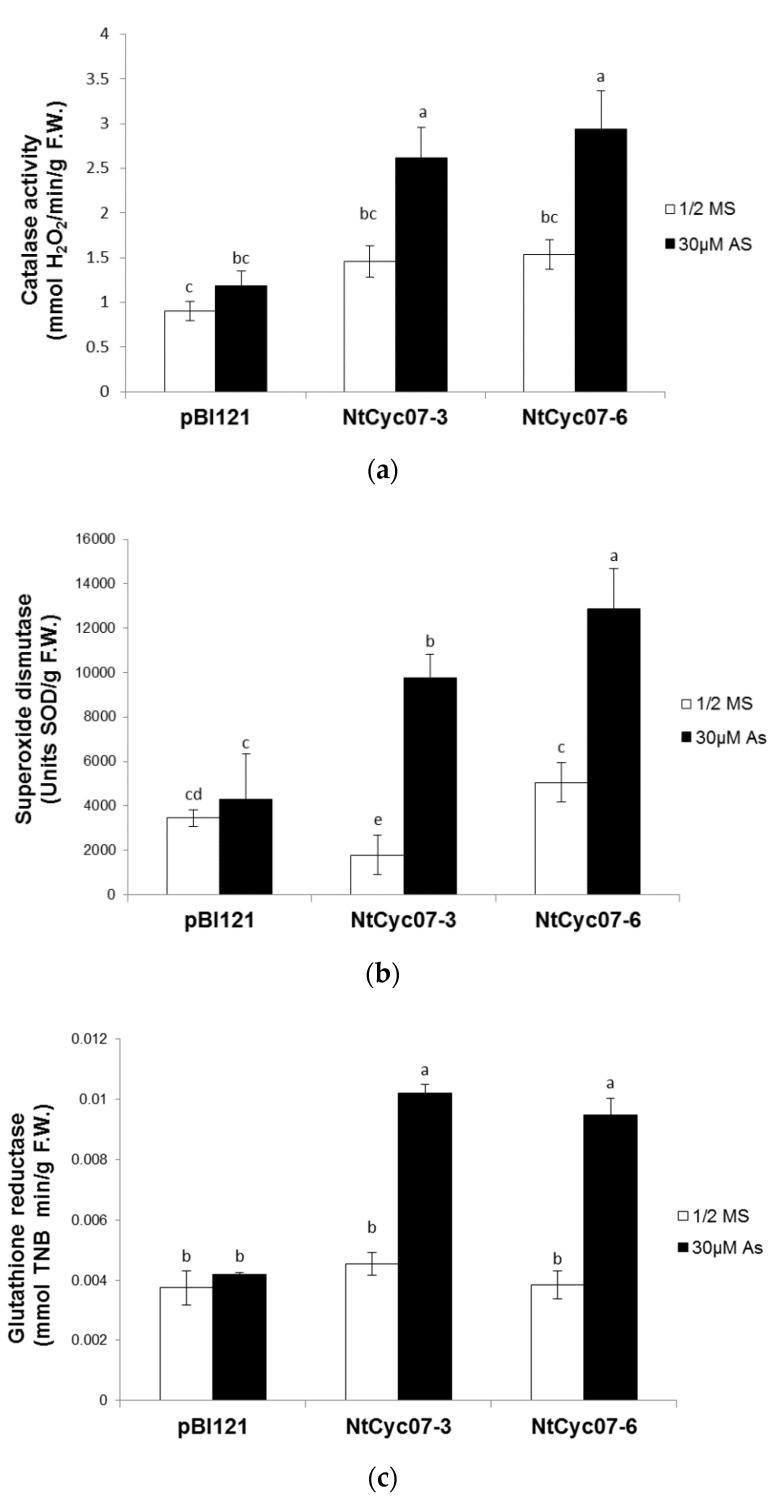
Activities of antioxidant enzymes in response to As(III) in *NtCyc07*-expressing tobacco. Activities of (**a**) catalase, (**b**) superoxide dismutase and (**c**) glutathione reductase in control (pBI121) and *NtCyc07*-tobacco. Plants were germinated and grown for 3 weeks on 1/2 MS agar media without or with 30µM As(III). 1/2 MS indicates a control (no As(III) treatment). The data are averages of three independent experiments per each treatment, and error bars indicate standard errors (S.E.). Different letters over columns indicate significant differences (*p* ≤ 0.05) between treatments.

In general, antioxidant enzymes ameliorate oxidative stress; therefore, the activities of catalase (CAT), superoxide dismutase (SOD) and glutathione reductase (GR) were examined (Figure 4). While the activities of all three enzymes were enhanced by As(III) in *NtCyc07*-tobacco, those of control plants were not induced, leading to the higher activities of antioxidant enzymes in *NtCyc07*-tobacco than in control plants. Regarding the lower As(III) level and higher activities of antioxidant enzymes in *NtCyc07*-tobacco, the higher antioxidant enzyme activity may be ascribed to the lower content of As(III). However, it is also possible that the activity of antioxidant enzyme is enhanced by *NtCyc07* itself, in addition to the lower As(III) content. Based on these results, it appears that the lower level of oxidative stress in *NtCyc07*-expressing tobacco under As(III) stress is ascribed to the higher activities of antioxidant enzymes and the lower level of As(III), when compared with control plants.

In support of our results, it has been reported that plants with reduced oxidative stress/higher activities of antioxidant enzymes show enhanced metal tolerances. Exogenous application of methyl jasmonate increased a Cd tolerance by reducing Cd-induced oxidative stress in rice [48]. Trehalose treatment increased a Cu tolerance by reducing oxidative stress in rice [49]. The overexpression of tobacco *PIC1* (Permease In Chloroplast 1) in tobacco enhanced Cd tolerances and activities of antioxidant enzymes [38]. Application of maleic acid increased Cr tolerances and activities of antioxidant enzymes and reduced oxidative stress in *Brassica juncea* [50]. Transgenic tobacco overexpressing tobacco *UBC1* (Ub-conjugating protein 1) displayed enhanced Cd tolerance and reduced oxidative stress [42]. Overexpression of tobacco *UBQ2* (Ub-extension protein 2) in tobacco and Arabidopsis increased Cd tolerances and reduced oxidative stress levels [45]. Overexpression of tobacco *Hb1* (non-symbiotic class 1 hemoglobin 1) increased Cd tolerances and decreased oxidative stress in Arabidopsis [40]. Transgenic *Arabidopsis* overexpressing miRNA156 displayed higher metal tolerance, lower ROS level and higher activities of antioxidant enzymes [46].

Regarding how NtCyc07 modulates expressions of *NIPs* and *PIPs* and activities of antioxidant enzymes, it is postulated that NtCyc07 may interact with transcription factors for *NIPs*, *PIPs*, and genes of antioxidant enzymes. This may be supported by the report that NtCyc07 interacts with ACR1 which is a transcription factor of *ACR3* (arsenite exporter) in yeast [33]. In addition, it cannot be excluded that NtCyc07 may modulate protein amounts and activities of transporters and antioxidant enzymes.

## 3. Materials and Methods 

### 3.1. Plants

In this study, we used the previously reported transgenic tobacco (*Nicotiana tabacum*) lines which overexpress a vector only (*pBI121*) and *NtCyc07* (NtCyc07-3 and -6) after harvesting new seeds [34]. Tobacco seeds were sterilized, vernalized, germinated, and grown for 3 weeks on half-strength Murashige and Skoog medium (MS medium, pH 5.7) with or without 30 µM sodium arsenite. The plates were placed in a growth chamber under a 16 h light (cool white fluorescent light at 150 mmol/m^2^/s) / 8 h dark photoperiod and 23/21 °C of day/night temperatures.

### 3.2. As(III) Tolerance and Accumulation

Arsenite tolerance was measured as described in the previous paper [34]. The As(III) tolerance rate (%) was calculated by dividing the fresh weights of the As(III) treated plants (*n* = 30) by the fresh weights of the control plants. To analyze the As(III) accumulation, control (pBI121) and transgenic (NtCyc07) tobacco seedlings grown for 3 weeks on agar media containing 30 µM As(III) were harvested, washed three times with ice-cold 5 mM CaCl_2_ and dried for 72 h at 60 °C. The dried sample (1 g) was digested with concentrated HNO_3_ and HClO_4_ in a Teflon Digestion Vessel (Savillex, Eden Prairie, MN, USA). The As(III) concentration was measured in triplicate using Inductively Coupled Plasma-Atomic Emission Spectroscopy (ICP-AES, Perkin Elmer Optima 4300 DV, San Diego, CA, USA) at a wavelength of 188.98 nm at The National Instrumentation Center for Environmental Management (NICEM, Seoul, Korea).

### 3.3. Quantitative Real Time PCR (qRT-PCR)

RNA isolation, cDNA synthesis, and qRT-PCR were performed as previously described [51]. Total RNA was isolated from 3 week-old 100 mg seedlings using gDNA removal column and RNA binding column in IQeasy^TM^ Plus plant RNA extraction mini kit (iNtRON biotechnology, Seongnam, Korea). cDNA was synthesized from 2 µg of total RNA using NEXscript^TM^ RT 2X master mix Oligo dT (NEX Diagnostics, Seongnam, Korea). Quantitative RT-PCR was performed using a CFX Connect™ Real-Time PCR Detection System (Bio-Rad Laboratories Inc., Hercules, CA, USA). Each 20 µL reaction mixture contains 10 µL of SYBER Supermix (SsoAdvanced™ Universal SYBR® Green Supermix, Bio-Rad, Hercules, CA, USA), 7 µL of nuclease-free water, 1 µL of cDNA, and 1 µL of gene specific primers. The qRT-PCR reaction conditions were 95 °C for 30 s, 40 cycles at 95 °C for 15 s, and 60 °C for 20 s. Experiments were performed in triplicate (three biological repeats), and each run contained three technical replicates for the cDNA and each primer set. Relative transcript levels were normalized to the internal quantitative control *NtActin* (ACCESSION #U60495.1), and the relative expression level of each gene was calculated using the 2-*ΔΔ*Ct method [52]. The gene-specific primers are presented in Supplementary Materials Table S1, and the MIQE check list for qRT-PCR is presented in Supplementary Materials Table S2.

### 3.4. Analysis of Antioxidant Enzyme Activity

#### 3.4.1. Sample Preparation

Leaf (0.2 g) of tobacco seedlings grown for 3 weeks on 1/2 MS agar media with or without 30 µM As(III) was ground in liquid nitrogen, homogenized in 1.2 mL of potassium phosphate buffer (0.2 M, pH 7.8) containing 0.1 mM EDTA, and centrifuged for 20 min at 15,000× *g*, 4 °C. Then, the supernatant was stored at −80 °C until using it for the enzyme assay. 

#### 3.4.2. Measurement of Antioxidant Enzyme Activity

Catalase (CAT; 1.11.1.6) activity was measured as previously described [53]. The reaction was initiated by adding 15 μL of leaf extract to 2.5 µL H_2_O_2_ (30% solution) prepared in 1 mL 50 mM of potassium phosphate buffer (pH 7.5) in a final volume of 1 mL. The extinction coefficient of H_2_O_2_ (40 M^−1^ cm^−1^ at 240 nm) was used to calculate the enzyme activity (millimoles of H_2_O_2_ per minute per gram fresh weight).

Activity of superoxide dismutase (SOD; EC 1.15.1.1) was determined using the modified NBT method [54]. The assay mixture contained 50 mM phosphate buffer (pH 7.8) with 2 mM EDTA, 1 mM riboflavin, 9.9 mM L-methionine, 0.025% Triton-X100 and 55 μM NBT. After adding 40 μL of leaf extract to 2 mL of reaction mixture, the reaction was started by illuminating the reaction mixture with a fluorescent lamp (15 W) for 10 min. The absorbance of the reaction mixture was measured at 560 nm. The reaction mixture without the leaf extract was used as a control. The enzyme activity (per gram fresh weight) of the sample was calculated based on the standard curve of pure SOD.

Glutathione reductase activity (GR; EC 1.8.1.7) was determined as described by Smith et al. [55]. The reaction mixture (1 mL) contained 10 µL of leaf extract, 100 mM potassium phosphate buffer (pH 7.5), 0.1 mM NADPH, 0.75 mM DTNB (2-nitrobenzoic acid), and 1 mM GSSG (glutathione disulfide). The reaction was initiated by addition of GSSG, and the absorbance at 412 nm was measured after 3 min when DTNB was reduced to TNB (2-nitro-5-thiobenzoate). The extinction coefficient of TNB (14.15 M^−1^ cm^−1^) was used to calculate the GR activity (millimoles of TNB per minute per gram fresh weight). All enzyme experiments were performed in triplicate, and each enzyme activity was measured three times.

### 3.5. Measurement of Oxidative Stress

Hydrogen peroxide (H_2_O_2_), superoxide (O_2_^−^) and malondialdehyde (MDA) were quantified in tobacco seedlings grown for 3 weeks on 1/2 MS agar plates as previously described [56,57,58]. For hydrogen peroxide measurement, 0.3 g of leaf was ground in liquid nitrogen, homogenized with 5 mL of 0.1% trichloroacetic acid (TCA), centrifuged for 15 min at 12,000× *g*, 4 °C, and the supernatant was collected. The reaction mixture contained 0.5 mL of leaf extract supernatant, 0.5 mL of 10 mM potassium phosphate buffer (pH 7.0), and 1 mL of 1 M potassium iodide. The same reaction mixture lacking the leaf extract was used as a blank. The concentration of H_2_O_2_ was determined based on a standard curve of known concentrations of H_2_O_2_. 

To measure the concentration of O_2_^−^, 100 mg of leaf was ground to a fine powder in liquid nitrogen, homogenized in 2 mL of 50 mM potassium phosphate buffer (pH7.8), and centrifuged for 10 min at 4 °C, 10,000× *g*. Then, 1 mL of the supernatant was mixed with 0.9 mL of 50 mM potassium phosphate buffer (pH 7.8) and 0.1 mL of 10 mM hydroxylamine hydrochloride, and then incubated for 20 min at 25 °C. Subsequently, 1 mL of 17 mM sulfonilamide and 1mL of 7 mM α-naphthylamine were added to the incubation mixture, further incubated under the same condition, and the absorbance was measured at 530 nm. The generation of O_2_^−^ was calculated based on a standard curve of NO_2_^−^ (nitrite). 

For the measurement of MDA, 0.5 g of tobacco seedlings was ground in liquid nitrogen, homogenized with 1.5 mL of 20% (*w*/*v*) TCA, and centrifuged for 5 min at 10,000× *g*. 2 mL of thiobarbituric acid solution (0.5% (*w*/*v*) in 20% TCA) was added to 1 mL of the supernatant, incubated at 95 °C for 15 min, quickly cooled in an ice bath, and centrifuged at 12,000× *g* for 10 min. The absorbance of the supernatant was measured at 450, 532 and 600 nm. The concentration of MDA was determined according to the following formula: concentration (μmol L^−1^) = 6.45 × (OD_532_ − OD_600_) − 0.56 × OD_450_ (OD: optical density). For all measurements, three independent experiments were performed. 

### 3.6. Visualization of H_2_O_2_ and Superoxide (O_2_^−^)

H_2_O_2_ was visually detected in tobacco leaves as previously described [59]. Leaves of 3-week-old plants grown on 1/2 MS agar media were immersed and infiltrated under a vacuum with 1.25 mg mL^−1^ 3,3’-diaminobenzidine (DAB) (D8001, Sigma-Aldrich, St. Louis, MO, USA) staining solution (pH 3.8), and incubated at 25 °C for 8 h. Subsequently, stained leaves were bleached in acetic acid-glycerol-ethanol (1/1/3) (*v*/*v*/*v*) solution for 5 min at 100 °C, preserved in glycerol-ethanol (1/4) (*v*/*v*) solution, and photographed. H_2_O_2_ was visualized as a deep brown color due to DAB polymerization. For each experiment, at least 10 plants were examined per line, and three independent experiments were performed.

O_2_^−^ (superoxide radical) was visually detected by nitro blue tetrazolium (NBT) (N6639, Sigma-Aldrich) staining using the modified method [60]. Briefly, leaves were detached and vacuum-infiltrated for 15 min in 1 mg mL^−1^ NBT solution prepared in 10 mM potassium phosphate buffer (pH 7.8) containing 10 mM NaN_3_ (sodium azide), incubated for 3 h at 25 °C, bleached as described above, and photographed. The production of O_2_^−^ was visualized as a blue color due to NBT precipitation. For each experiment, 10 plants were examined per line, and three independent experiments were performed.

### 3.7. Statistical Analysis

Two-way ANOVA was used to analyze the data using SAS software (version 9.1). The means were separated by Tukey’s multiple comparison test with significant differences at *p* ≤ 0.05.

## 4. Conclusions

1. The overexpression of NtCyc07 enhances As(III) tolerance by decreasing As(III) accumulation through the increased expression of putative As(III) exporter *NIP1;1*, *PIP1;1*, *PIP1;5*, *PIP2;1*, *PIP2;2*, and *PIP2;7*, and the reduced expression of putative As(III) importer *NIP3;1*, *NIP4;1*, and *XIP2;1*.
2. *NtCyc07*-tobacco displays lower levels of oxidative stress and higher activities of antioxidant enzymes. The lower oxidative stress in *NtCyc07*-tobacco may be attributed to the higher activities of antioxidant enzymes and the lower level of As(III). The higher activities of antioxidant enzymes in *NtCyc07*-tobacco may be ascribed to the lower content of As(III) and probably the direct effect/function of *NtCyc07*.

## Supplementary Materials

The following are available online at https://www.mdpi.com/2223-7747/9/11/1480/s1, Table S1: List of the primer sequences used in this study, Table S2: The MIQE check list for qRT-PCR.
