# Supplementary material for: Mechanism for Higher Tolerance to and Lower Accumulation of Arsenite in NtCyc07-Overexpressing Tobacco
Source: Plants (Basel). 2020 Nov 3;9(11):1480. doi: 10.3390/plants9111480 (PMC7692962; doi:10.3390/plants9111480)
Supplement: Supplementary file 1 [file plants-09-01480-s001.zip › plants-901627-resubmission-supplementary/Supplementary table 1_0929.pdf]

Table S1. List of the primer sequences used in this study.

Gene name	Accession	Primer Sequences	Amplicon size(bp)
NtActin	U60495.1	F: 5′-TGCTGATCGTATGAGCAAGG-3′<br>R: 5′-ATCCAGACACTGTACTTTCTC-3′	97
NtNIP1;1	XP_016487110.1	F: 5′-CGTTTCTGGTGTCGCAACTG-3′<br>R: 5′-TCCTTGCTGGGTTCATCGAC-3′	130
NtNIP2;1	XP_016451246.1	F: 5′-TCGCCACTGATACCAAAGCT-3′<br>R: 5′-GTCGTTGCTAGCCATTGCAG-3′	146
NtNIP3;1	XP_016460638.1	F: 5′-AGGGGCCATATTTGGTGCTT-3′<br>R: 5′-TCCACCCTTCACTACACTTGC-3′	149
NtNIP4;1	XP_016486634.1	F: 5′-GGTGGATGCAGGCCAATTTC-3′<br>R: 5′-CCCGCTAGAGTTCCGATGAC-3′	128
NtPIP1;1	NP_001313131.1	F: 5′-TCATTTGGCCACCATCCCAA-3′<br>R: 5′-AGCAAGAGCAGCTCCAATGA-3′	142
NtPIP1;5	CAA04750.1	F: 5′-ACCATTCCCATCACCGGAAC-3′<br>R: 5′-CAGCAAGTGCAGCTCCAATG-3′	133
NtPIP2;1	AAL33586.1	F: 5′-CCTGCTAGGACCTTTGGAGC-3′<br>R: 5′-CTGCGGAAAGAACCCAATGC-3′	164
NtPIP2;2	NM_001325404.1	F: 5′-AGCTAGAAGTTTTGGAGCTGCT-3′<br>R: 5′-GCTTTGACTGCTCCAGCTCT-3′	144
NtPIP2;7	NP_001313061.1	F: 5′-CTTTGTCGGAGCGTTGGTTG-3′<br>R: 5′-TCAAGTGCTGGGGTTGTTGT-3′	100
NtPIP2;17	NP_001312464.1	F: 5′-TGTCCCTGTATTGGCACCAC-3′<br>R: 5′-CACGGCTGCTCCAAAACTTC-3′	115
NtXIP1;1α	NP_001312796	F: 5′-TGGCTTCCAATGCTAGTCATGT-3′<br>R: 5′-GCCTTTGTGCCACTGTGAGA-3′	147
NtXIP2;1	XP_016489264.1	F: 5′-TCCCACTACAACATTTTCATGCA-3′<br>R: 5′-CGATATTGAGTAGTTTCGCGCA-3′	130
