# Supplementary material for: Mechanism for Higher Tolerance to and Lower Accumulation of Arsenite in NtCyc07-Overexpressing Tobacco
Source: Plants (Basel). 2020 Nov 3;9(11):1480. doi: 10.3390/plants9111480 (PMC7692962; doi:10.3390/plants9111480)
Supplement: Supplementary file 1 [file plants-09-01480-s001.zip › plants-901627-resubmission-supplementary/Supplementary table 2_0929.pdf]

Table S2. MIQE check list report.

| Experimental design | Check | qPCR oligonucleotides | Check |
|---|---|---|---|
| Definition of experimental and control groups | Y | Primer sequences | Y |
| Number within each group | Y | RTPrimerDB identification number | N/A |
| Assay carried out by the core or investigator's laboratory? | Y | Probe sequences | N/A |
| Acknowledgment of authors' contributions | Y | Location and identity of any modifications | N/A |
| Sample | | Manufacturer of oligonucleotides | Y |
| Description | Y | Purification method | Y |
| Volume/mass of sample processed | Y | qPCR protocol | |
| Microdissection or macrodissection | N/A | Complete reaction conditions | Y |
| Processing procedure | Y | Reaction volume and amount of cDNA/DNA | Y |
| If frozen, how and how quickly? | Y | Primer, (probe), $Mg^{2+}$, and dNTP concentrations | Y |
| If fixed, with what and how quickly? | N/A | Polymerase identity and concentration | Y |
| Sample storage conditions and duration (especially for FFPE[2] samples) | Y | Buffer/kit identity and manufacturer | Y |
| Nucleic acid extraction | Y | Exact chemical composition of the buffer | Y |

| | | | |
|---|---|---|---|
| Procedure and/or instrumentation | Y | Additives (SYBR Green I, DMSO, and so forth) | Y |
| Name of kit and details of any modifications | Y | Manufacturer of plates/tubes and catalog number | Y |
| Source of additional reagents used | Y | Complete thermocycling parameters | Y |
| Details of DNase or RNase treatment | Y | Reaction setup (manual/robotic) | Y |
| Contamination assessment (DNA or RNA) | N/A | Manufacturer of qPCR instrument | Y |
| Nucleic acid quantification | Y | qPCR validation | |
| Instrument and method | Y | Evidence of optimization (from gradients) | N/A |
| Purity ($A_{260}/A_{280}$) | Y | Specificity (gel, sequence, melt, or digest) | Y |
| Yield | Y | For SYBR Green I, $C_q$ of the NTC | Y |
| RNA integrity: method/instrument | Y | Calibration curves with slope and *y* intercept | Y |
| RIN/RQI or $C_q$ of 3′ and 5′ transcripts | Y | PCR efficiency calculated from slope | Y |
| Electrophoresis traces | Y | CIs for PCR efficiency or SE | N |
| Inhibition testing ($C_q$ dilutions, spike, or other) | N | $r^2$ of calibration curve | Y |
| Reverse transcription | | Linear dynamic range | Y |
| Complete reaction conditions | Y | $C_q$ variation at LOD | Y |
| Amount of RNA and reaction | Y | CIs throughout range | N |

| | | | |
|---|---|---|---|
| volume | | | |
| Priming oligonucleotide (if using GSP) and concentration | Y | Evidence for LOD | Y |
| Reverse transcriptase and concentration | Y | If multiplex, efficiency and LOD of each assay | N/A |
| Temperature and time | Y | Data analysis | |
| Manufacturer of reagents and catalogue numbers | Y | qPCR analysis program (source, version) | Y |
| $C_q$s with and without reverse transcription | N | Method of $C_q$ determination | Y |
| Storage conditions of cDNA | Y | Outlier identification and disposition | Y |
| qPCR target information | | Results for NTCs | Y |
| Gene symbol | Y | Justification of number and choice of reference genes | Y |
| Sequence accession number | Y | Description of normalization method | Y |
| Location of amplicon | N | Number and concordance of biological replicates | Y |
| Amplicon length | Y | Number and stage (reverse transcription or qPCR) of technical replicates | Y |
| In silico specificity screen (BLAST, and so on) | Y | Repeatability (intraassay variation) | Y |
| Pseudogenes, retropseudogenes, or other homologs? | N | Reproducibility (interassay variation, CV) | Y |
| Sequence alignment | Y | Power analysis | N |

| | | | |
|---|---|---|---|
| Secondary structure analysis of amplicon | N | Statistical methods for results significance | Y |
| Location of each primer by exon or intron (if applicable) | Y | Software (source, version | Y |
| What splice variants are targeted? | N/A | $C_q$ or raw data submission with RDML | N |

Y: Yes, We checked and qualified.
N: No, We did not perform or analyze this step
N/A: Not applicable
